# Incidental genomic findings in large scale research: using the “3-I framework” to reveal policy considerations

**DOI:** 10.3389/fgene.2025.1603420

**Published:** 2026-01-20

**Authors:** Suzanne Maria Onstwedder, Carla Van El, Wendy Rodenburg, Adrian Thorogood, Martina Cornelia Cornel, Tessel Rigter

**Affiliations:** 1 Department of Public Health Genomics and Screening, Centre for Health Protection, Dutch National Institute for Public Health and the Environment (RIVM), Bilthoven, Netherlands; 2 Section Community Genetics, Department of Human Genetics, Amsterdam UMC, Vrije Universiteit Amsterdam, Amsterdam, Netherlands; 3 Personalized Medicine Programme, Amsterdam Public Health Research Institute, Amsterdam, Netherlands; 4 The Terry Fox Research Institute, Vancouver, BC, Canada

**Keywords:** genetic testing/ethics, genomics, human genetics/ethics, human genetics/organization and administration, human genetics/standards, incidental findings, policy

## Abstract

**Introduction:**

Incidental findings (IF) can be yielded in genomic research that analyzes data from healthy participants or patients. The need for clear IF policies grows along with the increase in international genomic research, data collection and sharing efforts. This study aims to inform policy discussions and decisions about IF.

**Methods:**

We interviewed key stakeholders involved in Canadian, European, or international research projects. We used the 3-I framework for interview design and analysis, which distinguishes interests (i.e., agendas), ideas (i.e., values), and institutions (i.e., policy structures, e.g., laws) as factors that impact policy decisions. We integrated the three bioethical principles into this framework: respect for persons, beneficence, and justice.

**Results:**

Interviewees were from Canada (n = 7) and Europe (n = 4). Different IF policies are followed and practiced. Policy decisions are impacted by varying interests, ideas, and institutions. Prioritization of distinct interests and ideas varies between policies. Key policy considerations are: determining whose interest is prioritized; determining what is of best interest to the participant; determining who is responsible for what when research and healthcare institutional frameworks create tension; determining what is just and a fair distribution of benefits and burdens between individuals and populations; and determining how scarce time and money should be allocated.

**Discussion:**

Explicating policy considerations can help to further discuss and decide how IF policies will impact not only research participants, but also patients, citizens, professionals, the public and diverse populations. Technologies, cultural values, and laws and regulations evolve over time. Therefore, continuous discussions should be held.

## 1 Introduction

The scientific community has witnessed a rise in large scale genomics research projects. Many countries have set up their own national genomics data initiatives, accompanied by several international genomics projects (Onstwedder et al., 2022; European Commission, 2023). Often, these projects aim to ultimately improve health of both patients and citizens (Smetana and Brož, 2022). The large scale of these genomics data collections and exchange efforts raise policy questions, especially when different policies and practices are applicable in the distinct regions and countries involved.

Genome sequences are generally generated for a specific purpose in a specific setting, both in the clinic as well as research. With appropriate informed consent, these clinical and research data may also be shared and further analyzed in other research projects. Analysis in these projects may yield unexpected results that might be of clinical importance for research participants, both patients and citizens. Although different interpretations of incidental findings (IF) exist, we define it as an unsought genomic finding of potential medical relevance that is not related to the original purpose of the test, sometimes also called unsolicited findings. These findings can occur both in research as well as in clinical care. Of note, knowledge of the genome is still expanding, therefore it may not always be possible to definitively classify a finding as unrelated to the clinical phenotype under investigation (Hehir-Kwa et al., 2015; Vears and Amor, 2022). With the expected increasing use and reuse of genomic data in research (Byrd et al., 2020; Rehm et al., 2021; Kumuthini et al., 2023), jurisdictions are facing a need to make clear IF policies.

Guiding statements and policy recommendations with regard to IF have been drawn up by several professional organizations, such as the American College of Medical Genetics and Genomics (ACMG) (Miller et al., 2021), the European Society for Human Genetics (ESHG) (Van El et al., 2013), the Canadian College of Medical Geneticists (CCMG) (Boycott et al., 2015), and EuroGentest (Matthijs et al., 2016). These documents demonstrate a variety in international views and guidelines. Furthermore, regional and international differences exist in how these recommendations are translated into policy and practices. Reviews of policies and practices of incidental findings in genomic research found a support for the return of clinically actionable findings or findings with serious health conditions, but this was mostly based on views from representatives of Northern America (Ewuoso, 2016). Comparatively, the ESHG called for a more prudent approach, stating that an approach that avoids the yield of incidental findings is preferred. (van El et al., 2013). Furthermore, a lack of agreement in guidance for the return of incidental genomic findings results was described, as well as a need to establish a clear direction on how to develop more harmonized guidance across countries (Knoppers et al., 2015). The need for clear policies grows along with the increase in international data collection and sharing efforts. In order to develop effective IF policies, it is important to explore what considerations encourage or create tension in policy decisions, and where choices are needed.

**FIGURE 1:**
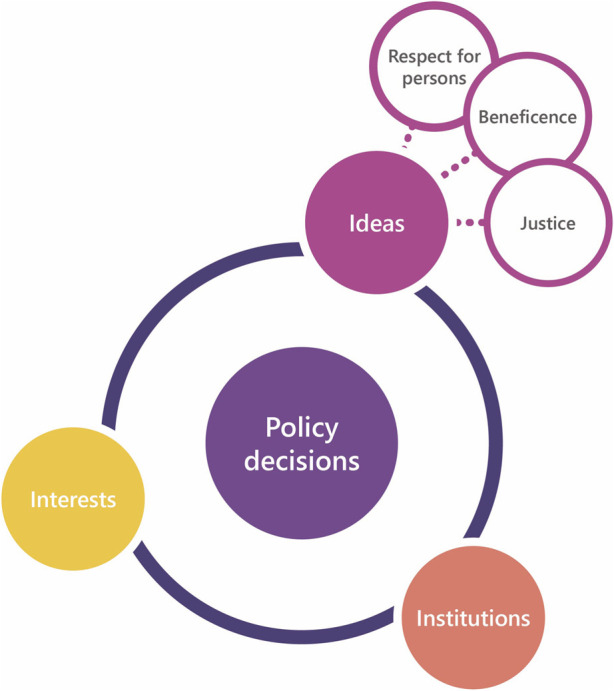
3-I Framework, as used in the current study. The 3-I framework distinguishes three elements that help to understand how or why past policies are created, and whether and how enacting policy change may be encouraged or challenged: interests, ideas, and institutions. In this study, the three bioethical principles are specified as values among ‘ideas’. Of note, the bioethical principles may also impact interests and institutions. Adapted from Gauvin, 2014.

Policy decision making can be studied using designated frameworks. The analytical ‘3-I framework’ helps to unravel policy considerations. It distinguishes three elements to understand how or why policies were created, and whether and how enacting policy change may be encouraged or challenged (Gauvin, 2014; Cernat et al., 2022). These three elements are *ideas*, *interests,* and *institutions*, see Figure 1 (Gauvin, 2014). *Ideas* refer to the values and beliefs of stakeholders regarding a policy issue, and the evidence and knowledge surrounding that issue. They are also described as “knowledge or beliefs about what is (e.g., research knowledge), views about what ought to be (e.g., values), or combinations of the two” (Pomey et al., 2010, p709). In this study, we interpreted ideas as norms, values and beliefs. *Interests* describe the agendas of various stakeholders. It reflects a common assumption that various stakeholders drive policy developments and choices by their interests, by a desire to influence policy, and by the impact of the relationships between the parties involved. Lastly, *institutions* include current and past policy structures that influence policy development, such as laws, regulations, governing organizations, and policy networks.

Looking at *ideas*, multiple values and beliefs might play a role in IF policy decisions in genomics research. The National Academies of Sciences, Engineering, and Medicine (NASEM) in the United States debated in 2018 that researchers are “ethically obligated to return urgent, medically actionable research results to their participants” and discussed that results should be returned in a way that “accommodates the full spectrum of community needs and preferences, regardless of participant’s social or economic status” (National Academies of Sciences and Medicine, 2018; Raymond et al., 2021). Yet, this raises several questions. Whether the return of medically actionable results to participants should be a responsibility of researchers is debated (Zawati and Knoppers, 2012). Furthermore, it is ambiguous whether and when returning findings accommodates the full spectrum of “community needs”. Especially in international research, participants are part of different social or cultural contexts with, e.g., varying healthcare systems and cultural or personal views on values as trust, privacy, or autonomy. Therefore, to allow for further analysis we chose to specify the ideas concept by differentiating three bioethical principles that describe the ethical norms of performing research (National Commission for the Protection of Human Subjects of Biomedical and Behavioral Research, 1979):

1. *Respect for persons,* which incorporates at least two ethical convictions: first, that individuals should be treated as autonomous agents, and second, that persons with diminished autonomy are entitled to protection.
2. *Beneficence*, which entails two general rules as complementary expressions of beneficent actions in this sense: first, do not harm and second, maximize possible benefits and minimize possible harms.
3. *Justice*, a fair distribution of burdens and benefits between individuals and populations within society.

Although the bioethical principles are here included as specifications of ‘ideas’, it is important to note that they may also impact interests and institutions, and *vice versa*.

The aim of this study is to inform policy discussions and decision making about IF in large scale genomics research. Therefore, we interviewed experts in large scale genomics research about *ideas, interests,* and *institutions* that impact current and future policy decisions about incidental genomic findings. Furthermore, we explored the bioethical principles and other ethical values that underly ideas.

This study answers the following research question:

- RQ1: what are key policy considerations when it comes to incidental genomic findings in large scale genomics research?

To answer this question, we first aim to broadly map and grasp the different aspects and arguments (ideas, interests, and institutions) that impact policy decision making. Then, we investigate what considerations encourage or create tension in policy choices, and where decisions are needed. Subsequently, we provide recommendations on how to move forward with these considerations in IF policies and policy discussions. For this, the following sub questions are answered:

- SQ1: what are important ideas, interests, and institutions when it comes to IF policy decisions?
- SQ2: where are ideas, interests, and institutions overlapping or conflicting, and create a challenge when making IF policy decisions?

## 2 Methods

This study was part of the European network staff eXchange for integrAting precision health in the health Care sysTems consortium, also called the ExACT project, which aims to build and exchange knowledge, staff, and network in precision medicine. We performed multidisciplinary expert interviews to obtain perspectives and experiences regarding IF policies and practices in genomics research. The COREQ Checklist was used to guide the reporting of the methodology and the results (Tong et al., 2007).

### 2.1 Interview participants

The interviewed experts were involved in either policy development or genomic research. We selected experts via purposive sampling. In order to determine which expertise we needed, we used a policy brief from the 1 Million Genomes (Rebers et al., 2023) to review and select experts representing the different roles of stakeholders and large scale genomics research projects. Then, we strived to include experts who were part of well-known organizations, and took part in relevant activities and research projects about IF in genomics research and return of results. Additionally, we invited experts with knowledge about the ethical, legal, and societal implications (ELSI), and patient representatives in genomics research.

Selected experts were from Canada or Europe, reflecting a wide variety of ethical, social and legal viewpoints. Yet, these regions typically do not take in inherently different standpoints on the return of IF, as, for example, the United States and Europe, which otherwise could lead to a more black and white reflection of policy decisions. Additionally, the inclusion of experts from both Canada and Europe reflected the ongoing collaboration between the research teams and institutions in these regions, as can be seen in the ExACT project, supporting the recruitment process and enhancing the study’s cross-regional relevance.

We invited experts via e-mail through the direct network of the authors and snowballing. The interviewed experts did not receive any form of compensation.

### 2.2 Data collection

We developed an interview guide based on the 3-I framework (Supplementary Material S1). Interviews were semi-structured. At the start of the interviews, the interviewer (SO) provided a small introduction about her personal background, followed by study background and aims. As discussed, different interpretations and definitions of IF exist. The interviewer did not provide a definition of IF at the start of the interviews, but openly asked what definition or interpretation the interviewed experts themselves have, and from there perspectives and considerations for policy were explored.

Interviews were performed between May and November 2023, lasted around 60 min, and were performed online via Microsoft Teams in English (n = 10) or Dutch (n = 1). They were performed by a researcher with training and prior experience in performing qualitative interview studies (PhD candidate, SO). Upon written consent (n = 10) or verbal consent (n = 1), the interviews were recorded and transcribed using the automatic transcription tool of Microsoft Teams and corrected by the researcher (SO). The experts were asked to check and correct for transcription errors. Their feedback was further processed prior to data analysis (SO).

### 2.3 Data analysis and interpretation

Transcribed files were analyzed with MaxQDA, version 2022 and 2024 (software updated during analysis). The analysis process and codebook were based on elements of the 3-I framework (Supplementary Material S1). Ideas were further divided over the bioethical principles. Ideas not directly linked to the three bioethical principles, e.g., knowledge generation, were coded as ‘other idea or value’. Three interviews were coded by two authors (CE and SO, or WR and SO), eight interviews were coded by one author (SO). The codebook was iteratively assessed, discussed, and further refined upon inter-coder discussions (authors CE, WR, SO). Additional codes were included: “barriers, facilitators, and prerequisites”, “dilemma’s and considerations”, “experts” definitions of incidental findings’, and “most important topic of the interview” (a routine interview question). During data analysis, the first author (SO) marked and selected findings or sections from the interviews for co-author discussions. The authors held regular 1 h discussions to collaboratively interpret the interview data, explore complexities, and debate and assess emerging interpretations. These discussions helped to gain understanding in considerations that contain arguments for or against specific policy choices (e.g., conflicting interests or ideas), which revealed where decisions are needed.

We included quotes from the interviews to illustrate important findings. Dutch quotes were translated into English. The quotes included in the manuscript were forwarded to the interviewees to check for correct interpretation and unidentifiability. Their feedback was processed (SO). SO took the lead in writing the manuscript. All authors provided critical feedback and helped shape the research, analysis and manuscript.

## 3 Results

In the following sections, we will first provide a description of the interview participants. Secondly, we will describe important ideas, interests, and institutions in light of IF policies identified during the interviews (SQ1). Lastly, the results will outline five challenging areas for policy where ideas, interests, and institutions are overlapping or conflicting (SQ2). Altogether, these results will indicate key policy considerations when it comes to incidental genomic findings in large scale genomics research.

### 3.1 Description interview participants

Interviewees were experts from Canada (n = 7) and Europe (n = 4; Austria, Belgium, France, and the Netherlands) and involved in (international) large scale genomics research projects, and/or representatives from organizations or initiatives with a role in policy development. They represented perspectives from data holders (n = 1), data users (n = 2), data subjects or representatives (n = 2), data returners (n = 2), and were experts with knowledge about ELSI (n = 4) (Rebers et al., 2023).

Interviewees’ professions were (multiple roles are possible): researchers (n = 8), laboratory experts (n = 2), genetic counselors (n = 2), patient representatives (n = 2), experts trained in ELSI (n = 4), and reviewers in ethical or institutional boards (n = 3). The experts had experiences ranging from 9 to 25 years (mean of 15,8 years, n = 10). Furthermore, their experience in their current role ranged from 1,5 to 15 years (mean of 9,4 years, n = 10). One expert did not provide information about their years of experience in their field and current role.

### 3.2 Ideas, interests, and institutions

Oftentimes, ideas and interests of specific stakeholders overlapped in analysis, e.g., health benefit as idea, as well as an interest of research participants. Therefore, they are discussed together below. The stakeholders with interest in corresponding ideas are specified in Table 1.

**TABLE 1:** Bioethical principles with corresponding ideas and interested parties in policy decisions about incidental genomic findings, listed by the bioethical principles.

Bioethical principle	Ideas^a^	Stakeholders with interest	Quote
Respect for persons	Autonomy and informed decision	Participant	*“from both clinical and research, […] we’re very careful about, like spending some time to accompany them through that decisions [return of results, … ] when it’s not, there’s not an active decision making, it makes the patient less engaged in thinking about this possibility.”*
Beneficence	Health improvement	Participant	*“And so there’s certainly added value in knowing about things that are preventable or treatable”*
	Prevent harm, e.g., anxiety or worry	Participant	*“You don't want to return anything to people that might create high anxiety for no reason”*
	Safeguarding validity of results and interpretation	Participant, researcher	*“There’s a lot of potential harm that can come from returning research results to providers and participants if it’s not done well. And there’s all those issues of, you know, overestimating the analytical validity of the results, misinterpreting the clinical validity of the results”*
	Determining and ensuring actionability	Participant, researcher, healthcare professional	*“Because medically actionable results[…], they’re only in theory actionable if someone actually takes action on them. Yeah. So in order to clinically action something, the channels to the clinic need to be created in order for those to, for those benefits to be realized”*
Justice	Fair distribution of research output	Public, policy	*“But in general, people who are offered research are people who tend to be English speaking, who have the time to sit down, to talk to somebody, to actually go to an appointment and then get that opportunity to be in research. So you do have a bit of a bias in terms of people that end up getting put into research projects. And because of that, if they are now jumping the queue somewhat because they’re getting research genomes that then push them into the clinical realm, then are they really getting the same access to care compared to people that don't get to be in research projects?”*
	Fair usage of healthcare resources	Healthcare professional, public, policy	
Other idea or value	Reciprocity	Participant	*“Another argument what is mentioned […] is reciprocity, huh, so I participate in research and then I should expect something in return, which is that you keep me informed of any findings […] as far as I’m concerned, solidarity should actually be a more important reason to participate in research”*
	Solidarity	Participants, public	
	Knowledge generation	Researcher, public	*“I think all the biobanks that are happening where they’re trying to collect samples and stuff like that, we’re just gonna find out so much more about the causes of different diseases”*
	Trust in research	Researcher	*“You don't want to return anything to people that might […] hurt the credibility of the research endeavor or trust in this, in these initiatives”*
	Transparency	Participant, public	*“You can really see that the value of transparency, for example, is becoming more and more important in society. So where 20 years ago you could say, well, we won't return it back, now it’s going in a different direction”*

^a^ Norms, values, and/or beliefs. Ideas and interested parties were sometimes directly stated by the experts, and otherwise interpreted by the authors.

#### 3.2.1 Ideas and interests

Stakeholders have their own ideas about and interest in IF and genomics research (Table 1, and Supplementary Material S1). These stakeholders include: participants-patients, participants-citizens, researchers, healthcare professionals, policymakers, and the public. Here, the public is viewed as both healthy citizens and patients who are not participants in genomics research that could receive IF. Ideas and interests from participants, both patients and citizens, were included by experts representing their views.

Interviewees discussed the following ideas and interests of participants:

• Respect for persons: autonomy and informed decision making about IF;
• Beneficence: health improvement, generating valid results and interpretation of IFs, and determining and ensuring actionability of IFs;
• Other idea: reciprocity.

Ideas and interests of other stakeholders, including researchers, healthcare professionals, policymakers, and the public, are expressed by the interviewees in terms of:

• Justice: a fair distribution of research output, fair usage of healthcare resources;
• Other ideas: solidarity, knowledge generation as a result of performing research, trust in research, and transparency.

Policymakers are assumably interested in all aspects, but were mostly mentioned in the context of societal and health system decision making. Table 1 shows that multiple parties can have similar ideas, i.e., norms, values, or beliefs. Yet, the reason why this is of their interests might differ. For example, participants, researchers, and healthcare professionals have interest in determining the actionability and utility of an IF: to improve their health (participant), to determine whether or not a genetic finding is actionable and improve their understanding of a disease (researcher), and to improve quality of care (healthcare professional).

#### 3.2.2 Institutions

Different institutions can support or enforce operationalization of above mentioned ideas and interests of different stakeholders by providing regulatory frameworks and implementing policies. To date, there are only few laws and regulations specifically applicable for IF in genomics research. Yet, multiple policies or regulations were mentioned that apply to elements of genomics research and IFs. Examples are the Declaration of Helsinki, genetic non-discrimination acts, General Data Protection Regulation (GDPR), and (proposed legislation for) the European Health Data Space (EHDS). Regulations are further supplemented by clinical or research guidelines such as the guideline for clinical practice (ICH-GCP; international council for harmonization, good clinical practice), and FAIR-principles for data management (findable, accessible, interoperable, and reusable) which inform and may increase the reuse of research data. Interpretation and implementation of these laws and policies differ between jurisdictions on national level, regional level, and institutional level. Respondents did not further explicate the perspectives of the different institutions currently providing guidance.

Experts reported the need for uniform and acknowledged guidelines that advise on what to return, how to return, and when. Mentioned relevant policy networks and organizations are the ACMG, CCMG, ESHG, American Society of Human Genetics (ASHG), EuroGentest, the World Health Organization (WHO), National Health Service (NHS) England, and the United States National Center for Biotechnology Innovation (NCBI). Some of these networks published guidelines about incidental genomic findings. As can be seen in Table 2, most recommendations have been written to inform clinical diagnostics and laboratories about genomic IF. Management of genomic IF in large scale research settings may call for additional considerations and challenges. These will be explored in the following sections.

**TABLE 2:** Published guidelines about incidental genomic findings.

Organization	Topic	Recommendation	Authors
American college of medical genetics and genomics (ACMG)	Incidental and secondary findings in clinical exome and genome sequencing	Recommends that laboratories performing clinical sequencing seek and report mutations of specified classes or types in a defined set of genes considered medically actionable, even when unrelated to the primary medical reason for testing. ACMG updates this gene list annually	Green et al., 2013; Miller et al., 2021
Canadian college of medical genetics and genomics (CCMG)	Genome-wide sequencing of germline DNA in the context of clinical genetic diagnosis	Does not endorse the intentional clinical analysis of disease-associated genes other than those linked to the primary indication, until the benefits of reporting incidental findings are established	Boycott et al. (2015)
EuroGentest	Diagnostic next-generation sequencing	Recommends that the analysis pipeline of diagnostic laboratories should focus on the gene panel under investigation in order to avoid the chance of secondary findings, and be validated accordingly. Furthermore, laboratories should provide information on the chance of unsolicited findings	Matthijs et al. (2016)
European society of human genetics (ESHG)	Whole-genome sequencing in healthcare	Recommends that, when in the clinical setting either targeted sequencing or analysis of genome data is possible, it is preferable to use a targeted approach first in order to avoid unsolicited findings or findings that cannot be interpreted	Van El et al. (2013)

### 3.3 Five challenging areas for policy: overlapping and conflicting ideas and interests in policy decisions

Interviewees discussed many ideas and interests that overlap or conflict regarding IF in large scale genomics research. Experts sometimes described them as dilemmas or policy considerations, other times the authors interpreted them as such. Five considerations were identified as key during the authors’ discussions.

#### 3.3.1 Determining whose interest is prioritized when all of them are at stake

The act of generating and returning IF in large scale genomics research holds several considerations. Many experts discussed the weighing of this decision on an individual level: is it beneficial for the participant? Yet, when thinking about this on a larger scale, the question rises whether this decision should and could still be made per individual (maximize health benefit, i.e., beneficence), or on a population level (promote fair allocation of societal resources, i.e., justice) (Table 3, quote 1 & 2)?

**TABLE 3:** Quotes that describe policy considerations about determining whose interest is prioritized.

Policy considerations	Quote
Whose interest is prioritized when all of them are at stake	*1. “if we’re just talking about research, […] there has to be some balance with respect to achieving the research goals which is collective in nature and incremental developments to benefit science and all people, and weigh that up against the individual benefits of returning result to individual participants of that said research project.”*
	*2. “I see it as a scientific imperative, but I also see it as an ethical imperative when done properly, of course, in a way that protects participants, protects their choices and advances science and allows people to benefit […] from science.”*
	*3. “suppose the citizen says no, and you find a genetic variant of which you think, yes, this is really serious, I sleep badly as a researcher that I can’t give that to that patient, huh, then again there might be reasons for us to overrule that, so to speak. So well, it’s all complicated.”*

Furthermore, the interests of professionals such as healthcare professionals and researchers are also at stake. For example, the professional duty to return life-saving information can conflict with a participants’ decision to not receive any findings, and can create tension among or between the involved researchers or healthcare professionals (quote 3).

Here, the interests of involved parties, including participants, professionals, and populations are colliding, and prioritization of one of these above the other will impact policy decisions.

#### 3.3.2 Determining what is of interest to the participant, and who will decide this

Autonomy and informed decision making are important when it comes to generating and returning IF. Experts discussed generally two current practices. The first one is an autonomy-focused approach in which participants should be their own agents, i.e., respect for persons (Table 4, interpreted from quote 4). To achieve this, participants should receive all the necessary information to make an informed decision before giving consent. This includes, e.g., the criteria used for selection of what results to return (e.g., validity and actionability).

**TABLE 4:** Quotes that describe policy considerations about determining what is of interest to the participant.

Policy considerations	Quote
What is of interest to the participant, and who will decide this	*4. “I don't think it’s fair to not allow people the opportunity to receive those types of findings if they want them, but they need to be informed about what that might mean and have the opportunity to opt out if they don't want them.”*
	*5. “Like with all these things, it’s a risk benefit calculation, so you would be weighing the potential benefits to the participant.”*
	*6. “do you then actually as a researcher have a duty to protect the data as best you can by throwing away that key [connecting research data to participants], or do you as a researcher have a duty to give that patient that feedback of those side findings and therefore working with a file that still has that key. That’s quite an interesting dilemma.”*
	*7. “most of the time, actionability is defined from a clinical point of view from a medical point of view. I’m not sure actionability is always defined from a patient’s perspective or from a parent’s perspective”*

Yet, providing all this information could create decisional burden for the participants. Participants may not always be fully aware or able to comprehend and weigh all the factors to make a decision. Therefore, some experts are in favor of a more paternalistic approach: researchers and healthcare professionals make decisions in line with participants’ suggested interests, and balance the pros and cons for the participant (quote 5 & 6). Considerations that help in weighing this decision from a clinical and health system perspective are analytical and clinical validity–are the results valid–and actionability–are preventative or treatment options available. Yet, utility and actionability from a patients’ perspectives could also be considered (quote 7).

Decisions about generating and returning IF to participants balance respect for persons and beneficence. So here, policy decisions are impacted by conflicting ideas that steer towards two different approaches.

#### 3.3.3 Who is responsible for what when research and healthcare institutional frameworks create tension

Experts often mentioned that the yield and return of IF to participants blurs the boundaries between research and care. This blurring can lead to multiple issues (Table 5, quote 8).

**TABLE 5:** Quotes that describe conflicting institutional frameworks and policies of research vs. healthcare.

Policy considerations	Quote
Who is responsible for what when research and healthcare institutional frameworks create tension	*8. “trying to draw the line between those two things [clinical laboratory testing and research testing] is challenging, but I think also important. […] There’s the conflict of interest, where often you have the research investigators also the treating physician. We talk about the action bias that comes from that. So the researcher wants to find meaningful results and the treating physician wants to have actionable outcomes for their patients and you combine those two things at one person and you get a tendency to overinterpret the significance of a finding.”*
	*9. “the question from a public health perspective then becomes are we investing the money to then have to clinically validate something that we see on a research basis or should it actually be put back in the researchers budget to then say because you’ve now found this, that you should be responsible then for doing the Sanger sequencing [clinical validation, … ]. Because there’s many times where the research diagnosis actually ends up being the diagnosis for the child, and we treat research almost like it’s the clinical gold standard.”*
	*10. “I definitely do not see the patients in the center. Um, I see the sales pitch of we can make the healthcare system cheaper when we introduce early detection thanks to genomics and genetics, and therefore we will build up those systems.”*
	*11. “There are emerging examples of health systems that are using genomic screening, or genetics as a first line test for patients that are presenting and they are finding a significant number of actionable results in those contexts as well.”*
	*12. “Other reason for not doing it [returning results] is it’s not the researcher’s job actually, right? The researcher gives general-level results that are relevant to the general population, but not necessarily to the individual participant”*

Interviewees acting in both research and healthcare mentioned a gap between quality standards in research and healthcare. Research laboratories often lack the clinical accreditation that a healthcare laboratory requires. As a result, clinicians need to order confirmatory genetic testing if they want to act on IFs. Spending healthcare resources to validate research IFs may displace patients with clinical needs (quote 9). This raises questions about justice.

Additionally, the ESHG poses that the return of findings that are found opportunistically requires a cautious approach. They state that proportionality should be ensured, also balancing healthcare benefits and resources (justice) (De Wert et al., 2021). Moving forward, they propose that setting up pilots could help to generate data about the impact, feasibility, and proportionality of the opportunistic return of genomic findings. Genomics research can be seen as a way to screen, induce early detection, and reduce healthcare costs, depending on the clinical utility of the finding at hand (quote 10 & 11). While literature on the economic evaluation of return of IFs is limited, it does not yet prove to be cost-effective (Fontes Marx et al., 2021).

To limit healthcare expenses, the responsibility to perform clinical confirmation of IFs could be transferred to research. This improves the validity of research results, but also increases costs. However, it may not directly fit with the role of a researcher (quote 12), and can further blur the boundaries between research and healthcare.

The blurring boundaries between research and healthcare, distinct regulations that are applicable for either, and subsequent implications for roles, responsibilities and future policy decisions fit within the 3-I framework subsection *institutions*.

#### 3.3.4 What is just: how do we ensure a fair distribution of benefits and burdens between individuals and populations

There has been an uneven representation of diverse populations in genomics research. Less scientific evidence for gene-disease causing variants from underrepresented populations is available. Because of this bias, less meaningful IF may be generated and returned to underrepresented populations. As a result, they will be less likely to benefit from research (Table 1, quote “Justice”) and interpretation of IF will require more caution (Table 6, quote 13).

**TABLE 6:** Quotes that describe policy considerations about a fair distribution of benefits and burdens between individuals and populations.

Policy considerations	Quote
What is just: how do we ensure a fair distribution of benefits and burdens between individuals and populations	*13. “we can’t necessarily be very certain about whether we’re finding false positives in some of these cultures that are underrepresented because the databases just don't have enough information about them. […] So I think they’re probably needs to be a bit more caution about what we are returning”*
	*14. “So in [name country], maybe we value the idea of privacy and you know, confidentiality and also transparency. There may be other centers that are not so democratic that may value, you know, standardization and you know, for the public good and so forth. There’s that cultural layer on top of that*
	*15. “But with regard to the general public, rare disease, people living with rare diseases are more, tend to be more, uh, willing to participate in research and to share their data”*
	*16. “Because if you just go in with that sort of mindset of I need to get this from the community, it’s just reinforcing sort of historical colonial sort of practices that many of these communities have experienced, […] without them receiving any sort of benefit. So I try to be mindful of those sort of historical harms that communities have experienced.”*
	*17. “Power is productive and when we draft policies, whether it’s an incidental findings policy, whether it’s healthcare policy, it has to be driven by values. What do we want to achieve at the very end? […] it needs to be carefully weighed out, again, striving for this balance of enabling research and protecting. […] and it will always be a tough balancing act that we never have, or maybe for brief moment in time.”*

Many interviewees mentioned ensuring equity as key topic. Key to realizing a fair distribution of burdens and benefits between individuals and populations (i.e., justice) is including diverse populations or subgroups in genomics research. Experts mentioned the need for informed consent procedures that fit with the needs of diverse populations, funding to enable this research, and researchers’ ability to engage with diverse and hard to reach populations. Furthermore, cultural history, disease history, views, and values differ between populations and subgroups (quote 14 & 15). Therefore, it is key to explore and include them in IF policies so that it also serves their values and benefits them (interpreted from quote 16). Here, the principle of justice and interests of distinct populations will impact future policy decisions.

Additionally, the diversity in current IF policies creates concerns in terms of justice: participants from institute or region X may receive IF, while participants form institution ore region Y may not. Moving forward requires reflection on the impact of reported policy considerations and decisions. Policies regarding generation and analysis of research data, and generation and return of IF will impact։ a) who has access to the knowledge, b) who benefits and who experiences the burdens, and c) the subsequent power provoked (quote 17). This emphasizes the significance of effective and timely policies that enable an appropriate and balanced distribution of benefits, burdens, and power when science advances internationally. Furthermore, it points to the relevance of representation from the diverse interests that are at stake at the institutions that offer policy guidance.

#### 3.3.5 Making and implementing explicit policy decisions requires time, money, and infrastructure

Researchers and institutes hold the responsibility to maximize the benefits and minimize the harms of their actions (i.e., beneficence). Interviewees underlined the importance of having a plan before returning IF (Table 7, quote 18). This plan should include sufficient budget, analytics support, and personnel trained to return these results. Additionally, it is key that relevant information about participants is available via ICT systems. Apart from participants’ preferences to receive IF or not, other relevant participant information includes։ disease and family history (e.g., hereditary or familial disorders), a known diagnosis that fits the generated IF, age, and whether they are alive or deceased. An important consideration in *a priori* planning is the workload and resources of researchers. Increasing the tasks and responsibilities assigned researchers, e.g., confirmatory testing, impacts research budgets and output (quote 20).

**TABLE 7:** Quotes that describe that making and implementing policy decisions requires time, money, and infrastructure.

Policy considerations	Quote
Making and implementing explicit policy decisions requires time, money, and infrastructure	18. “*I think it’s a responsibility of both the researcher and institution that there has to be a plan and the resources behind that plan. The reason why we plan is so that it's well resourced, well thought out and the benefits are maximized and the harms or the costs are minimized.”*
	*19. “research is done usually with a specific timeline and specific budgets. And in order for us or for researchers to be able to do more, they will require more funding. […] because otherwise we would be losing the beneficial output that can come out of it.”*
	*20. “If they’re going from a researcher to a care team, that you have a committee or somebody who’s not in a conflict of interest actually, to think about, think about those results and whether they will potentially benefit the participant.”*
	*21. “it should not be up solely to a scientist who may not have the experience in deciding whether something is returnable to return it. So resources should be made available to help with that decision.”*

Furthermore, interviewees mentioned that researchers may not always have the right knowledge and expertise or may not feel confident to decide in which cases IFs are suitable to be returned to a participant (quote 18). And even when a researcher or expert is qualified to make this decision, some argued that it should not be the decision of one person alone. Involving a third person helps to improve outcome (quote 19), i.e., maximize benefits and minimize harms, and prevent conflicting interests, over-interpretation of results, or action bias. Interviewees mentioned research ethics boards or an equivalent of molecular tumor boards as potential advising third parties.

Here, the principle of beneficence and interests of participants and researchers overlap, and impact future policy decisions.

### 3.4 Key policy considerations regarding incidental genomic findings in large scale genomics research

**FIGURE 2:**
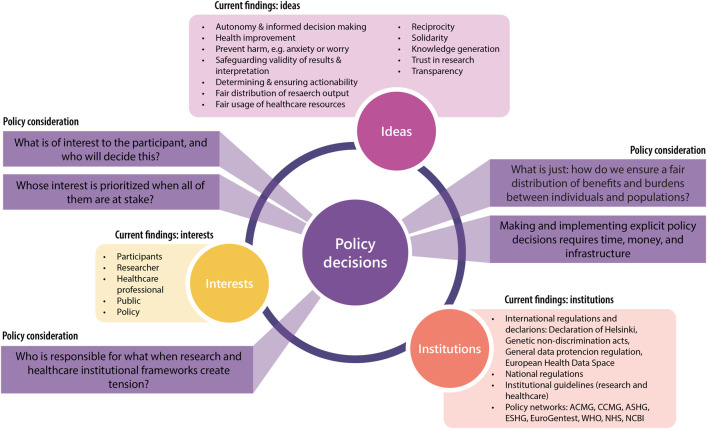
Visual summary of current findings and identified policy considerations. A visual representation of the ideas, interests, institutions, and policy considerations identified in this study. The authors have placed the policy considerations in the 3-I framework where they thought them most appropriate, but they may require incorporation of multiple aspects and therefore cannot be explicitly linked to one or two I’s. Adapted from Gauvin, 2014.

All combined, the identified ideas, interests, institutions, and policy considerations are depicted in Figure 2. We can interpret from this figure that policies about genomics IF could incorporate many aspects that extend beyond healthcare and research setting, and include societal and ethical considerations as well. The policy considerations point out that ideas, interests, and institutions may create tension among and between each other. When comparing this to the guidelines described in Table 2, it reveals that current policies focusing on genomics IF may fit within a broader societal and ethical reflection, especially considering that genomic data availability will continue to increase globally.

## 4 Discussion

Our study revealed various overlapping or conflicting ideas, interests, and institutions when it comes to incidental genomic findings in large scale genomics research. We found a heterogeneity in cultural views, national regulations and guidelines, and research practices. Because of this heterogeneity, specific recommendations or proposed actions to move towards policies may not be viable in all settings, e.g., large national projects or international collaborations. Yet, we could draw five policy considerations that would benefit from further discussions (Box 1). Below, we provide recommendations for policy networks on how to move forward with these considerations.

Box 1 **Points to consider for policy regarding IF in large scale genomics research.**

While the analysis in this study clarifies what interests of what stakeholders are at stake, it also shows that further decisions by policy makers should be explicitly made. To guide these policy decisions, we therefore propose these five “points-to-consider”.

- Whose interest is prioritized when all of them are at stake: participants, researchers, healthcare professionals, the public at large, and diverse populations will all be impacted by policy decisions.
- What is of interest to the participant, and who will decide: considering autonomy and informed decision making, what are benefits and harms, and how should they be maximized or minimized.
- Who is responsible for what when research and healthcare institutional frameworks create tension: blurring lines between research and healthcare creates tension between the roles and responsibilities of researchers and healthcare professionals.
- What is just: how do we ensure a fair distribution of benefits and burdens between individuals and populations: validity and utility of IF differ between individuals and populations because of e.g., knowledge bias and access to follow-up care, and IF policies and practices vary between jurisdictions.
- Making and implementing explicit policy decisions requires time, money, and appropriate infrastructures: maximize benefits and minimize harms are served by planning beforehand, and ensuring resources are available.

Many similarities can be found between the described application of bioethical principles in the interviews and how these principles are researched and described in literature. Examples of research on operationalization of the respect for persons in genomics research are studies looking into broad or dynamic consent (Dankar et al., 2020), or ethical considerations of children partaking in genomic research (Halley et al., 2023). Multiple statements from varying international associations illustrate how beneficence is weighed in decisions to return incidental genomic findings (Green et al., 2013; van El et al., 2013; Boycott et al., 2015; Matthijs et al., 2016; Miller et al., 2021). Furthermore, justice in genomics research has been described to be multifaceted. The field has paid much attention to the uneven representation of populations in research databanks. Currently, researchers attempt to restore this bias through initiatives that generate more knowledge about underrepresented populations (Popejoy and Fullerton, 2016), and incorporate their values into research. Meanwhile, some individuals or populations may experience an unfair distribution of outcomes, e.g., inequitable access to follow-up care and testing (Goering et al., 2008; Mersha and Abebe, 2015). The (in)justice of generating knowledge and returning incidental genomic findings by researchers therefore requires consideration of many aspects.

### 4.1 Recommendations for policy networks and discussions

As said, we found a heterogeneity in cultural views, national regulations and guidelines, and research practices. How this heterogeneity may impact the management of genomics IF in light of future international genomics research and data sharing efforts remains to be determined. Yet, it will be fruitful to openly discuss and exchange ideas about the areas we identified as challenging in policy making among countries and stakeholders that collaborate, analyze, and share datasets in this international field and have a role in policy making. Enabling these discussions will help to understand how different countries and stakeholders weigh and prioritize ideas, interests, and institutions, where their views match or differ, and to what extent we could benefit from policy adjustment or alignment. As a next step, countries could move towards towards formulation of shared principles regarding genomics IF in large scale research as a next step while having leeway to develop and adjust their own policies that are fitting to their national context.

Furthermore, it is essential to initiate broader discussions that move beyond viewing genomics IF solely from a healthcare or research perspective. We found that currently, research and healthcare frameworks and policies are intertwining with the management and return of genomics IF. By taking an overarching view, policy could better address the complex interaction between healthcare and research, and explore which responsibilities should be assigned to whom. Extending to an even broader approach, taking into consideration all different ideas and interests we identified, we should consider genomics IF within a wider societal and ethical context. By weighing the different ideas and interests at stake, and by reflecting on which impact is desired for society, policymakers could further develop policies and steer towards practices that not only look at the clinical or research impact for the participant and researcher, but also for society and the public.

Interviewees discussed several relevant networks to further discuss these policy considerations: ESHG, ASHG, CCMG, EuroGentest, and WHO. Key in discussing these policy considerations is having all the right people at the table. It will be important to bring in different perspectives about ‘what is a benefit and to whom’, ‘what is a fair distribution’, and ‘who should be responsible’. The following stakeholders should be given a voice to express their interest:

• data subjects, e.g., research participants–both patients and healthy individuals –,
• data users and data holders, e.g., researchers and healthcare professionals involved in the yield of IF,
• data returners, e.g., genetic counselors,
• parties involved in or responsible for research policies and decision making, e.g., research ethic boards and funding agencies,
• underrepresented populations.

### 4.2 Limitations

This study explores which aspects impact policy decisions about IF in genomics research, and which decisions are challenging and would benefit from further consideration and discussion. This study is limited in providing a complete overview of how ideas, interests, and institutions are currently weighed and prioritized. Furthermore, this interview study contains a relatively small sample size from selected regions. Therefore, it may be that not all aspects that impact policy decision making about genomics IF internationally are uncovered. Yet, providing a complete and extensive oversight on all potential perspectives regarding genomic IF policies and practices goes beyond the goal of this study. The policy considerations identified in this study highlight challenging areas about which guidelines are not yet clear and which will benefit from future policy discussions. Extending this exploratory research by incorporating more views from experts from, e.g., multiple European countries, the United States, Asia, and Australia, will help to unravel how much views and perspectives differ between countries, and to what extent shared policies and practices on the management of IF in genomics research are desirable and feasible.

### 4.3 Conclusion and future directions

Our study reveals five considerations for policy that would benefit from further discussions with multiple stakeholders. These discussions can help to inform policy discussions and decisions, by clarifying impacts on and perspectives of research participants, but also patients, citizens, researchers, healthcare professionals, the public and underrepresented populations. Discussions about weighing and prioritization of distinct interests and ideas could aid in making policy decisions that are viable within and across national boundaries.

This study looked at policy considerations, but did not prioritize these considerations. Furthermore, we interviewed experts from Europe and Canada. Gathering perspectives from other nations will further help in making decisions that are fruitful and effective in multiple countries. Policies will often be written with a specific goal in mind, e.g., to improve research outcomes or quality of care. Therefore, it will be relevant to evaluate if IF policies to date have served certain interests more than others, and include the voices and interests of all involved. Furthermore, it would be relevant to incorporate policy considerations about opportunistic screening. When genomic sequencing is performed on the complete genome, genomic secondary findings could be considered no longer incidental, but to be expected. The distinction between unexpected and expected findings is then difficult to make. However, it remains important to have clear policies describing when or what to return, and by and to whom.

Lastly, technologies, cultural values, and laws and regulations evolve over time. Therefore, continuous discussions should be fostered. Notably, policy decisions about IF in genomics research are part of bigger political discussions. The blurring of boundaries between research and healthcare extends beyond the field of genomics. Secondary usage of health data for research is being institutionalized at European level, e.g., in the context of the recently published regulatory framework European Health Data Space (EHDS) (Molnár-Gábor et al., 2022; Kari et al., 2024; European Parliament Council of the European Union, 2025). The legislation for EHDS is stated to be designed to benefit all EU citizens, including patients, healthcare professionals, researchers, policymakers, and industry (European Commission, 2025). It contains clauses on return of clinically relevant findings. Whether this will be the case for genomic IF, is still debated and will become apparent in its implementation in the coming years. Therefore, it will be beneficial to discuss issues and share insights and solutions in international initiatives in which researchers, healthcare professionals, and policymakers of different fields meet and collaborate.

## Data Availability

The datasets presented in this article are not readily available because participants only gave consent to publication of the data included in the article. Raw data cannot be anonymized because statements can be traceable to specific experts or expertise. Requests to access the datasets should be directed to the corresponding author.
