# Supplementary material for: Incidental genomic findings in large scale research: using the “3-I framework” to reveal policy considerations
Source: Front Genet. 2026 Jan 20;16:1603420. doi: 10.3389/fgene.2025.1603420 (PMC12863703; doi:10.3389/fgene.2025.1603420)
Supplement: Supplementary file 1 [file Supplementaryfile1.docx]

Supplementary Material 1

## Topic guide interviews

| *Nr* | *Question* | *Topic* |
| --- | --- | --- |
| ***Stakeholders and interests in genomics research*** | | |
| 1. | Can we begin by outlining your area of interest/expertise in genomics research?   1. Data user, data holder, data subject, medical professional, legal/policy expert | Interest, expertise |
| 2. | Could you describe the added value of genomics research to your field or work? | Added value of genomics research |
| 3. | Do you use genomic data in your work? If so, how and for wat purpose? | Aims and agenda |
| 4. | Are you part of a large genomics research initiative, which makes DNA-data available across borders? If so, what are the aims of this initiative? | Experience, international |
| ***Ideas regarding incidental findings*** | | |
| 5. | Can you please tell me about your views about or experience with return of results in genomics research? | Views, experience, broad |
| 6. | What do you see as incidental findings? | Definitions |
| 7. | What are your views about or experience with incidental findings? | Views, experience, incidental findings |
| 8. | What are arguments for or against reporting incidental findings?   - 1. How would you weigh the pros and cons? | Arguments, values |
| 9. | What are important requirements or considerations to report incidental findings according to you? | Requirements, considerations |
| 10. | What are your views on reporting back genomics results in international research initiatives? Are there additional requirements or considerations? | Views, requirements, international |
| ***Institutions surrounding incidental findings*** | | |
| 11. | How would you describe the current policies surrounding reporting incidental findings in your nation or institute? | Current policy |
| 12. | What are these policies based on, in terms of laws or regulations? | Laws, regulations, procedures |
| 13. | And internationally? | Laws and procedures, international |
| 14. | Which parties play a role in policy making and enforcement? What are their tasks? | Roles and tasks, policy making |
| 15. | Which parties play or should play a role in practices and procedures? What are their tasks? | Roles and tasks, policy performance |
| 16. | If so, are there any additional roles and tasks in international genomics research projects? | Roles and tasks, international |
