# Supplementary material for: Incidental genomic findings in large scale research: using the “3-I framework” to reveal policy considerations
Source: Front Genet. 2026 Jan 20;16:1603420. doi: 10.3389/fgene.2025.1603420 (PMC12863703; doi:10.3389/fgene.2025.1603420)
Supplement: Supplementary file 2 [file Supplementaryfile3.docx]

Supplementary Material 3

## Expanded textual description of key aspects with corresponding ideas and values and interested parties in policy decisions about incidental genomic findings.

Interviewees often discussed that participants should be viewed as **autonomous agents** and should be enabled to make **informed decisions** about which incidental findings they would like and would not like to receive. Varying informed consent procedures are carried out to enable this decision making process, including opt-in, opt-out, and dynamic consent procedures via written forms, private or group consultations, or online decision aids. Autonomy and informed decision making is based on respect for persons.

**Health improvement** was discussed by multiple experts as potential benefit of returning incidental genomic findings to participants. Returning IF may increase their knowledge about personal health risks, improve or personalize treatment, and prevent development of disease or limit the effects of a condition that could occur in the future. Providing IF to participants as a way of providing advantages in response to their collaboration to research, i.e. **reciprocity**, was also discussed. Opposed to reciprocity, the act of participating in research could also be based on **solidarity**. In this case, the public is seen as the party that benefits from people partaking in research instead of the research participant themself.

It is key to **prevent harm** among participants when results are returned, such as anxiety or worry after receiving results. This could be tackled by appropriate information provision and informed decision making prior to the return of results. Furthermore, the benefits and risks should be weighed carefully before returning findings. Safeguarding the **validity of results and interpretation**, and ensuring that the results are **actionable**, e.g. treatments and healthcare resources are available, can further prevent inflicting potential harm to participants. Safeguarding validity of results and interpretation and ensuring actionability also impacts the interest of researchers and healthcare professionals. Returning results of limited validity can reduce **trust in research**, which further impacts researchers. The decision to return incidental genomic findings in order to improve health, the importance of preventing harm, ensuring that the findings are valid and correctly interpreted, and actionability of a finding is well determined are based on beneficence.

Another key aspect that often came up during the interviews was a fair **distribution of research output**. To date, there is an uneven representation of diverse populations in genomics research, including a lack of diverse ethnic backgrounds, and people with a lower socio-economic status. Yet, individuals are expected to benefit from research conducted among people of comparable background. Improving diversity and inclusion in genomics research will yield a more equal distribution of research output. Once recruited for genomics research that may generate incidental findings, participants may receive the opportunity to receive these findings, and seek additional medical support. Subsequently, the **usage of healthcare resources** may be delegated to these participants. Combining this with the uneven representation of participants in genomics research results in an unequal distribution of research output and healthcare resources on population level. These two key aspects impact the value of creating and striving for justice among populations. These key aspects impact the interests of healthcare professionals, policy, and the public.

For researchers and the public, the act of **generating knowledge** when individuals partake in research is a fruitful impact. This may further help to understand the causes of varying diseases, including common complex diseases, as well as rare diseases about which not much is yet known and few cases have been found. As mentioned before, it is important for researchers to uphold the trust of the public and participants in research. Meanwhile, participants and the public have an interest in **transparency**, which may cause them to expect more from research in terms of return of results and communicating about new scientific insights.
