# Supplementary material for: Incidental genomic findings in large scale research: using the “3-I framework” to reveal policy considerations
Source: Front Genet. 2026 Jan 20;16:1603420. doi: 10.3389/fgene.2025.1603420 (PMC12863703; doi:10.3389/fgene.2025.1603420)
Supplement: Supplementary file 3 [file Supplementaryfile2.docx]

Supplementary Material 2

## Coding tree interview analysis

**Bold: Code group**

Underlined: Code

*Cursive: Sub code*

**Ideas**: the norms, values, and beliefs of stakeholders and the evidence and knowledge surrounding an issue.

- Respect for persons: which incorporates at least two ethical convictions: first, that individuals should be treated as autonomous agents, and second, that persons with diminished autonomy are entitled to protection.
- Beneficence: which entails two general rules as complementary expressions of beneficent actions in this sense: first, do not harm and second, maximize possible benefits and minimize possible harms.
- Justice: a fair distribution of burdens and benefits between individuals and populations within society.
  - *Procedural justice*
  - *Compensatory justice*
  - *Distributive justice*
- Other values/beliefs

**Interests**: the agendas of various stakeholders. 1) Who wins and who loses? 2) By how much do they win or lose?

- Participants’ interest
  - *Patients*
  - *Citizens*
- Researchers’ interest
  - *Specific researchers*
- Health professionals’ interest
  - *Specific HPs*
- Public interest
  - *Specific sub-populations*
- Policy interest
  - *Specific policy actors*
- Other

**Institutions:** are the current and past policies, laws, regulations, governing structures, and policy networks that influence policy development.

- Governing structures: national or international laws and regulations that describe, restrict and prohibit specific activities regarding research and incidental findings, as well as the mandate and accountability of the government and experts, such as the accountability of healthcare professionals in the decisions they make.
- Policy networks: which unite the government and professionals outside of the formal process of government, such as professional groups and associations.
- Policy legacies: past policies and their influence, which may make it difficult to deviate from the path created by previous choices and achieve policy reform.

**Barriers/facilitators/prerequisites**

**Dilemma/consideration**

**Definition of incidental findings**

**Most important topic of the interview**
